# Complete Absence of the Uterus

**Published:** 1851-08

**Authors:** 


*Complete Absence of the Uterus.*—The *Gazette Médicale* mentions the following case, which shows very forcibly, that we should always, in our attempts at enlarging the vagina, recollect that the uterus may be entirely absent. The patient married at thirty-two, but never menstruated, though she had now and then a discharge from the vagina; the mammæ and the rest of the body were well formed, but she had always been indifferent to coition. The woman died at fifty-seven, of phthisis, the lungs being saturated with tubercles. The labia majora and the clitoris were of full size, but the vagina so narrow that it hardly admitted the index finger; the vaginal canal was only one inch long, and ended in a cul-de-sac, behind which there was not the slightest trace of the uterus. The Fallopian tubes were situated in the broad ligaments, the latter being placed behind the bladder. The ovaries were found under the Fallopian tubes, the former being somewhat hardened, and containing in their interior compact little masses. The uterus was completely wanting, not the least rudiment of the organ being distinguisable. —*London Lancet.*

